# Hirschsprung disease at a tertiary hospital: Patient profile, management and outcomes

**DOI:** 10.4102/hsag.v30i0.2883

**Published:** 2025-06-24

**Authors:** Elizabeth Brits, Layla Moosa, Muhammad Kola, Osman Cassim, Zafeerah Khan, Rummanah Cajee, Aslam Salie, Muhammed Peer, Mohammed S. Hoosen, Joseph B. Sempa

**Affiliations:** 1 Department of Surgery, Faculty of Health Sciences, University of the Free State, Bloemfontein, South Africa; 2 Department of Biostatistics, Faculty of Health Sciences, University of the Free State, Bloemfontein, South Africa

**Keywords:** Hirschsprung disease, paediatric surgery, outcomes, surgical management, postoperative complications

## Abstract

**Background:**

Hirschsprung disease (HD), a congenital condition marked by absent ganglion cells in the colon, causes serious digestive problems. It affects 1 in 5000 newborns worldwide, predominantly males. Delayed diagnosis and limited resources influence outcome. The clinical profile, management, and outcomes of HD at Universitas Academic Hospital Complex (UAHC), a tertiary hospital in central South Africa, were investigated.

**Aim:**

The aim was to identify healthcare challenges and enhance patient care in a resource-restricted setting.

**Setting:**

UAHC, Bloemfontein, South Africa.

**Methods:**

A retrospective cross-sectional study of all 65 paediatric surgical patients treated for HD during 2010–2021 was conducted. Data extracted from electronic medical records were analysed regarding demographics, disease presentation, diagnostic methods, treatment approaches and postoperative outcomes.

**Results:**

A total of 65 patients, mostly male (83.1%), with a median age at diagnosis of 87 days, were analysed. Symptoms included abdominal distension and difficulty passing stool; 30.8% developed Hirschsprung-associated enterocolitis (HAEC). Diagnostic methods mainly used rectal suction biopsies. Surgery often required multi-stage procedures due to late diagnosis and extensive disease. Postoperative complications were common (e.g. ileus, surgical site infections, HAEC and hypomotility). Many patients needed additional surgeries. Follow-up indicated not only a high survival rate but also significant loss to follow-up and long-term complications.

**Conclusion:**

Managing HD in resource-limited settings presents major challenges, where late diagnosis and scarce diagnostic resources affect outcomes.

**Contribution:**

Managing HD is challenging because of delayed diagnosis, limited resources, complications and loss to follow-up, necessitating better perioperative care. Early diagnosis and structured follow-up can improve outcomes.

## Introduction

Hirschsprung disease (HD) is a congenital disorder marked by the absence of ganglion cells in the distal colon, leading to inadequate innervation and functional obstruction. This condition affects approximately 1 in 5000 live births, with a notably higher incidence among males than females (male-to-female ratio 4:1) (Lampus 2023; Szylberg & Marszałek 2014). The impact of the disorder on the quality of life is profound, with affected individuals often experiencing constipation, incontinence and enterocolitis, which significantly diminish their daily functioning (Saleem et al. 2023).

Delayed passage of meconium is often observed from the onset, typically within the first 24 h – 72 h postpartum (Lampus 2023). Infants may also present with constipation, poor feeding, inadequate weight gain and abdominal swelling. Sometimes, the initial symptom could be diarrhoea due to stool blockage. Diagnosis can be delayed until adulthood in mild cases, particularly in ultra-short segment HD, compounding the risk of severe complications, such as Hirschsprung-associated enterocolitis (HAEC), which may occur pre- or postsurgery (Lampus 2023).

Early medical consultation is advised to address these challenges effectively for symptoms such as delayed meconium passage or abdominal distension (Sosnowska et al. 2016). Diagnosis typically involves a detailed clinical assessment followed by confirmatory histopathological examination through rectal biopsy, determining the absence of ganglion cells (Pecoraro et al. 2021). The preferred treatment approach is surgical, often through a single-stage pull-through procedure, although more complex cases may require staged surgeries (Jain et al. 2023; Lampus 2023). Given the complexity of HD, long-term follow-up is crucial to monitor and manage potential complications such as constipation, incontinence, toxic megacolon and recurrent enterocolitis (Kyrklund et al. 2020; Sosnowska et al. 2016).

At the Universitas Academic Hospital Complex (UAHC) in central South Africa, managing HD (approximately six patients per year) presents unique challenges that reflect broader issues related to limited resources and healthcare access. These challenges significantly affect this complex condition’s diagnosis, treatment and outcomes. One of the primary concerns at UAHC is the impression of the clinicians of delayed presentation of the disease (diagnosis made after the neonatal period, specifically beyond the first year of life) (Bhargava & Khedkar 2024; Ostertag-Hill et al. 2024), which is commonly observed in other illnesses in this region (Arnold et al. 2019; Brisighelli et al. 2020; Brits & Le Grange 2023). Many patients present late because of socioeconomic barriers, cultural perceptions of symptoms and initial misdiagnoses as functional constipation. Such delays can lead to severe complications, including the high risk of HAEC, chronic obstruction and malnutrition (Brisighelli et al. 2020; Trinidad et al. 2022).

The limited availability of specialised diagnostic resources, such as manometry and adequate pathological services, exacerbates these issues, often necessitating reliance on less definitive clinical and radiographic assessments (Trinidad et al. 2022). Consequently, patients at UAHC frequently undergo multi-stage surgical procedures rather than the ideal single-stage pull-through operation. This approach is typically dictated by the extent of the disease and the patient’s condition at presentation, reflecting a compromised management strategy because of the delayed diagnosis (Saleem et al. 2023).

Postoperative care at UAHC also encounters significant challenges. The region faces high rates of postoperative complications and mortality due to the advanced state of the disease at presentation and the constraints in postsurgical follow-up and support (Trinidad et al. 2022). The complexity of managing postoperative complications, such as incontinence, constipation and toxic megacolon, is compounded by these factors, necessitating a robust long-term management strategy (Kyrklund et al. 2020).

Consequently, there is an urgent need for targeted research and healthcare interventions at UAHC and similar settings. This study aimed to understand the patient profile, disease presentation, treatment approaches and outcomes of HD at this facility to identify and address regional healthcare challenges and enhance patient care in a resource-restricted setting.

## Research methods and design

### Setting

The UAHC in Bloemfontein, Free State province, South Africa, provides paediatric surgical services to most of central South Africa, including parts of the Eastern and Northern Cape provinces and the neighbouring Lesotho.

### Study participants and measurement

This retrospective cross-sectional study encompassed all paediatric surgical patients who underwent treatment for HD by the Paediatric Surgical Department in UAHC, Bloemfontein from January 2010 to December 2021. There were no exclusion criteria. Data for this study were sourced from the MEDITECH electronic patient files. The data collected included demographic, diagnostic, treatment and outcome information.

### Validity

Standardised diagnostic criteria and histopathological confirmation via rectal biopsies reinforced internal validity. Construct validity was strengthened by multiple diagnostic methods, including biopsies and contrast enemas, with defined surgical outcomes. External validity was supported by including all HD cases from a tertiary centre, making findings relevant to similar settings (Murad et al. 2018).

### Reliability

For reliability, data extraction from MEDITECH (electronic files) minimised errors. A standardised collection protocol and pilot study ensured feasibility. Inter-rater reliability was improved through independent data verification (Alavi, Biros & Cleary 2022). Objective clinical indicators and established statistical methods further enhanced reproducibility.

### Pilot study

A pilot study was undertaken with the initial 10 patients to assess the data collection feasibility and the datasheet’s adequacy. No modifications to the data collection form were needed, and these patients were incorporated into the main study.

### Data analysis

Data were captured on a Microsoft 365 Excel spreadsheet (Microsoft Corporation; Redmond, WA, US) and analysed by the Department of Biostatistics, Faculty of Health Sciences, University of the Free State. Statistical analysis was conducted by using *R* (version 4.3.0; R Foundation for Statistical Computing; Vienna, Austria). Results were summarised by frequencies and percentages for categorical variables, and median and interquartile ranges (IQR) for numerical variables.

### Ethical considerations

Ethics approval was obtained from the Health Sciences Research Ethics Committee (HSREC) of the University of the Free State (reference No. UFS-HSD2022/0546/2908-0001) and the Free State province Department of Health. Patient information was anonymised, and no identifying details were documented. Data were stored on a password-protected computer to ensure data security. Because data were collected from archived patient records, informed consent was not required.

## Results

### Demographic variables and patient presentation

The study comprised the files of all 65 patients seen during the study period, with 83.1% (*n* = 54) being male and 81.5% (*n* = 53) being Black. The median age at diagnosis was 87 days (IQR 29, 534), ranging from 4 days to 4 704 days (12.9 years). As shown in Table 1, patients were predominantly from South Africa (*n* = 55; 84.6%), with more than half residing in the Free State province (*n* = 37; 56.9%). Patients from Lesotho constituted 15.4% (*n* = 10) of the cohort. All patients tested negative for HIV.

**TABLE 1:** Demographic information and clinical presentation of paediatric surgical patients who underwent treatment for Hirschsprung disease (*N* = 65).

Variables	*n*	%
**Sex**		
Male	54	83.1
Female	11	16.9
**Ethnicity**		
Black	53	81.5
White	2	3.1
Mixed race	10	15.4
**Origin**		
Free State province	37	56.9
Northern Cape province	17	26.2
Lesotho	10	15.4
Other	1	1.5
**Signs and symptoms**		
Abdominal distension	64	98.5
Difficulty in passing stools	63	96.9
Air trapping	57	87.7
Delayed passage of meconium	37	56.9
Forceful deflation	35	53.8
Vomiting	32	49.2
HAEC	20	30.8
Failure to thrive	17	26.2
Feeding intolerance	13	20.0
Bowel perforation	3	4.6
**Associated anomalies**		
None	55	84.6
Malrotations	0	0.0
Down syndrome	5	7.7
Cardiac lesion	7	10.8
Neurocristopathy	3	4.6

HAEC, Hirschsprung-associated enterocolitis.

Common presenting symptoms and associated anomalies are summarised in Table 1. Abdominal distension was the most common symptom (*n* = 64; 98.5%), followed by difficulty passing stool (*n* = 63; 96.9%) and air trapping (*n* = 57; 87.7%). Delayed passing of meconium occurred in 37 (56.9%) patients. Furthermore, 10 (15.4%) patients had associated anomalies, including cardiac lesions, Down syndrome and neurocristopathies.

### Diagnostic procedures and investigations

Rectal suction biopsies (*n* = 29; 44.6%) were the most common diagnostic procedure (Table 2). Repeat biopsies were performed in one-third of the cases (*n* = 22; 33.8%) necessitated by superficiality, distal placement or inconclusive results. Contrast enemas were conducted in 36 (55.4%) patients, revealing a transition zone (TZ) in 94.4% (*n* = 34/36) of cases, with correlation to the affected segment observed in 30 (88.2%) of these patients.

**TABLE 2:** Diagnostic procedures and investigations in paediatric patients with Hirschsprung disease.

Procedure	*n*	%
**Type of biopsy (*n* = 65)**		
Rectal suction biopsy	29	44.6
Full-thickness rectal biopsy	21	32.3
Open/laparoscopic biopsy	15	23.1
Need for repeat biopsies (*n* = 22) *		
Too superficial	20	91.0
Inconclusive	9	40.0
Too distal	8	36.4
Contrast enema (*n* = 36) *		
TZ observed	34	94.4
Reverse rectosigmoid ratio	29	80.6
Sawtooth appearance	13	36.1
TZ correlates with involved segment (*n* = 34)	30	88.2

TZ, transition zone.

* , Multiple options were possible; therefore, the *n*-values exceeded the total number of patients in these subgroups.

### Disease specifications and management

Results pertaining to specific characteristics of HD and management of patients are summarised in Table 3. Most patients (*n* = 51/64; 79.7%) were diagnosed with short-segment HD, while a minority had ultra-short or long-segment HD (14.1% and 6.2%, respectively). None of the patients had total colonic HD. Among those undergoing surgical repair, a two-stage procedure was performed in half (*n* = 31/63; 49.2%) of the patients. The laparotomy-assisted Swenson procedure was most commonly performed (*n* = 42/63; 66.7%). The Duhamel procedure was not performed in any of the patients (Table 3).

**TABLE 3:** Disease specifications and management of paediatric patients with Hirschsprung disease.

Disease specifications and management	*n*	%
**Segment (*n* = 64)**		
Short	51	79.7
Ultra-short	9	14.1
Long	4	6.2
Total colonic	0	0.0
**Surgical stages (*n* = 63)**		
One-stage	18	28.6
Two-stage	31	49.2
Three-stage	14	22.2
**Surgical procedure (*n* = 63)**		
Transanal pull through	12	19.0
Swenson (laparotomy-assisted)	42	66.7
Swenson (laparoscopically assisted)	1	1.6
Soave	8	12.7
Duhamel	0	0.0
Covering stoma (*n* = 63)	14	22.2
Initial/primary stoma (*n* = 64)	46	71.9
**Reasons for initial stoma placement (*n* = 46)**		
Very dilated proximal bowel	39	84.8
Unable to deflate with washouts	17	37.0
Done at a different institution	4	8.7
Bowel perforation	1	2.2
Long-segment HD	1	2.2

HD, Hirschsprung disease.

Initial stoma placement as part of a multi-stage repair was performed in over two-thirds of the patients (*n* = 46/64; 71.9%), mainly due to significant proximal bowel dilation (*n* = 39/46; 84.8%). Covering stomas were created in 22.2% (*n* = 14/63) of the patients, aligning with the number of patients undergoing three-stage repairs (Table 3).

### Postoperative course, complications and outcome

As shown in Table 4, approximately one-third of the patients (*n* = 19/63; 30.2%) required redo or additional surgery. Neorectal biopsies (*n* = 11/19; 57.9%) for recurrent symptoms were performed most commonly, followed by ostomy creation (*n* = 9/19; 47.4%). Short-term complications affected 61.9% of patients (*n* = 39/63), with postoperative ileus being the most prevalent (*n* = 7/39; 18.0%), followed by superficial wound sepsis (*n* = 6/39; 15.4%) (Table 4). None of the patients developed urinary retention, anastomotic leak or bowel ischaemia.

**TABLE 4:** Postoperative course and complications in patients with Hirschsprung disease (*N* = 65).

Postoperative course, complications and outcome	*n*	% of total group	% of subgroup
**Redo or other surgery (*n* = 19)**	-	30.2	-
Redo neorectal biopsies	11	-	57.9
Stoma	9	-	47.4
Redo pull-through	4	-	21.1
Adhesiolysis	4	-	21.1
Anal anastomotic stricturoplasty	3	-	15.8
Pelvic or abdominal abscess drainage	1	-	5.3
**Short-term complications (*n* = 39)**	-	61.9	-
Ileus	7	-	18.0
Wound infection	6	-	15.4
Sepsis	2	-	5.1
Pelvic abscess	2	-	5.1
Pneumonia	1	-	2.6
Other	15	-	23.8
**Long-term complications (*n* = 41)**	-	65.1	-
Enterocolitis	15	-	36.6
Hypomotile colon	15	-	36.6
Anastomotic stricture at anal level	8	-	19.5
Hypermotile bowel	6	-	14.6
Internal anal sphincter achalasia	4	-	9.8
Adhesive bowel obstruction	3	-	7.3
Ischaemic stricture of bowel	2	-	4.9
Anal canal injury	1	-	2.4
Overstretched external anal sphincter	0	-	0.0
Retained aganglionic segment	0	-	0.0
Acquired aganglionosis	0	-	0.0
Other	16	-	39.0
Lost to follow-up	18	27.7	-
**Alive or demised (*n* = 47)**	-	-	-
Alive	45	69.2	95.7
Demised	2	3.1	4.3

Long-term complications were observed in 65.1% of the patients (*n* = 41/63), with enterocolitis and colon hypomotility being the most frequent (both *n* = 15/41; 36.6%) (Table 4). None had an overstretched external anal sphincter, retained aganglionic segment or acquired aganglionosis as long-term complications. At the end of the study period, 18 (27.7%) of the initial 65 patients were lost to follow-up. Of those that were followed up, 95.7% were alive (*n* = 45/47) and 4.3% were confirmed deceased (*n* = 2/47) (Table 4). Of the total group, 69.2% were alive and 3.1% had demised. The median follow-up duration was 774 days (2.1 years) (IQR 349, 2031).

## Discussion

### Demographic variables and patient presentation

The study revealed a high proportion of male participants with HD (83.1%), with a male-to-female ratio of 5:1, similar to the global range observed in other low-resource setting studies (3.3–5.5:1) (Bandré et al. 2010; Bradnock et al. 2017; Chatterjee et al. 2021; Ongeti et al. 2009; Pecoraro et al. 2021). The median age at diagnosis was 87 days, which was earlier than typically seen in resource-limited countries (1–2 years) (Hailemariam et al. 2024; Mabula et al. 2014) and even better but more comparable to findings from the United States (118 days) (Pecoraro et al. 2021). The ideal time for diagnosis remains the neonatal period. The age range for diagnosis in this study varied widely from 4 to 4 704 days, suggesting significant diagnostic challenges and highlighting the necessity for healthcare providers to recognise the diverse clinical presentation of HD for timely intervention (Bandré et al. 2010; Hailemariam et al. 2024; Ongeti et al. 2009; Tan et al. 2022).

Common symptoms of HD included abdominal distension and difficulty passing stools, as similarly reported in the literature (Bandré et al. 2010; Gao et al. 2022; Lampus 2023; Min et al. 2021). Less typical symptoms such as vomiting, feeding intolerance and failure to thrive were also noted, indicating a diverse presentation of the disease (Beltman et al. 2022; Min et al. 2021). Additionally, the prevalence of HAEC in this study was 30.8%, which was within the global range of incidence rates (2.3% – 55%), while bowel perforation was a rare but severe complication observed in 4.6% of the cases, reflecting similar findings in other studies (3.6% – 7%) (Beltman et al. 2022; Gao et al. 2022; Mabula et al. 2014; Zhang et al. 2023).

The findings in this study (cardiac anomalies 10.8%, Down syndrome 7.7%) support existing literature on the prevalence of cardiac anomalies and Down syndrome in patients with HD, confirming the associations noted in previous studies (cardiac anomalies 12.3%, Down syndrome 2.0% – 15.0%) (Bandré et al. 2010; Cantone, Catania & Zulli 2023; Klein & Varga 2020; Szylberg & Marszałek 2014). The research also observed cases of rarer neurocristopathies (4.6%), such as Waardenburg syndrome and congenital central hypoventilation syndrome, which aligned with earlier research, underscoring HD’s connection with various genetic and developmental anomalies (Klein & Varga 2020; Szylberg & Marszałek 2014). These findings emphasise the need for comprehensive evaluation and management to improve patient care and outcomes by addressing HD and its associated conditions.

### Diagnostic procedures and investigations

Rectal suction biopsies were the preferred diagnostic method for HD in 44.6% of the cases. However, one-third (33.8%) of the biopsies required repetition, which was slightly higher than in other studies, including a South African study (5% – 26.1%) (Hartford et al. 2023; Neeser et al. 2024). This highlights the importance of adequate sample depth and not being too distal to avoid inconclusive results. Current guidelines recommend biopsy locations to be at least 2 cm above the dentate line (Jain et al. 2023). These findings reflect the diagnostic complexities of HD and the necessity for precise histopathological techniques to enhance patient care, with repeated biopsies often prompted by issues such as distal placement or thickened rectal mucosa in delayed presentations (Jain et al. 2023).

Contrast enemas were performed in over half of the patients and proved an effective screening tool, with a transitional zone identified in 94.4% of the cases. Furthermore, there was a high concordance rate with the identified TZ corresponding to the affected segment in 88% of the patients. This correlation was in keeping with reports in the literature, including South African studies, especially in short-segment diseases (44.4% – 88.5%) (Chen et al. 2017; Haikal et al. 2020; Msomi, Mangray & Du Plessis 2017). These findings underscore the reliability and usefulness of contrast enemas in screening for HD, which can significantly aid clinicians in guiding subsequent diagnostic and therapeutic interventions for improved patient outcomes (Msomi et al. 2017; Trinidad et al. 2022).

### Disease specifications and management

Approximately 80% of the cases in this study were classified as short-segment HD, confirming it as the most prevalent subtype globally (67.3% – 81.3%) (Bradnock et al. 2017; Hailemariam et al. 2024; Mabula et al. 2014; Saleem et al. 2023). The occurrence of both ultra-short (0.9% – 14%) and long-segment (3% – 22.2%) HD in this study (ultra-short 14.1%; long 6.2%) was consistent with their lower reported incidences (Bradnock et al. 2017; Hailemariam et al. 2024; Mabula et al. 2014; Saleem et al. 2023; Szylberg & Marszałek 2014). The absence of total colonic aganglionosis (which occurs in 2% – 13% of the cases) and skip lesions (23 cases reported in the literature) in this study reflected their rarity, as noted in the existing literature (Ahmad et al. 2021; Bradnock et al. 2017; Chang et al. 2021; Hailemariam et al. 2024; Mabula et al. 2014; Moore 2015; Moore, Sidler & Schubert 2013), highlighting the established patterns of HD subtypes and concurring with the findings of prior research.

Different surgical approaches in managing HD, with a two-stage procedure preferred in 49.2% of the cases, had been performed in this study cohort, reflecting literature, including low-resource settings, supporting this method to minimise operative stress while achieving favourable outcomes (36.5% – 98.4%) (Bradnock et al. 2017; Huerta et al. 2023; Munnangi et al. 2023; Oyania et al. 2024; Saleem et al. 2023). A one-stage procedure was implemented in 28.6% of the cases, aligning with other studies, including those from low-recourse settings, that confirmed its feasibility and effectiveness (0% – 60.5%) (Bandré et al. 2020; Félicien et al. 2024; Obermayr et al. 2009; Sosnowska et al. 2016; Zbaida, De Vos & Sidler 2021). Three-stage procedures in 22.2% of the patients indicated a more cautious strategy for complex cases, consistent with suggestions for multistage surgeries in scenarios involving late presentations, malnutrition or extensive disease (Giuliani et al. 2020; Sosnowska et al. 2016). These findings emphasise the necessity for tailored treatment plans and continuous research to improve surgical techniques and outcomes in HD management.

The laparotomy-assisted Swenson procedure, performed in two-thirds of the cases, remains a primary surgical method because of its historical significance and proven effectiveness, despite a trend towards less invasive approaches (Munnangi et al. 2023). Primary transanal pull-through procedures, although performed in only 19.0% of the cases in this study, represent the move towards minimally invasive techniques, even in low-resource settings, associated with benefits such as shorter hospital stays and favourable outcomes (Bandré et al. 2010; Félicien et al. 2024; Munnangi et al. 2023; Negash et al. 2022; Obermayr et al. 2009). Additionally, the frequent initial placement of stomas in cases with significant proximal bowel dilation reflects a cautious strategy, enhancing safety and outcomes in complex cases (Bandré et al. 2010; Bradnock et al. 2017; Huerta et al. 2023; Montalva et al. 2023; Munnangi et al. 2023). These practices underscore a balanced approach to surgical management in HD, combining traditional techniques with modern, less invasive methods and personalised care, and highlight the need for continued research and collaborative clinical decision-making to optimise surgical results.

### Postoperative course, complications and outcome

Redo or additional surgery was performed in 30.2% of the cases, similar to previous indications that many patients may need further interventions after initial surgery (Munnangi et al. 2023; Quiroz et al. 2020). Neorectal biopsies were the most common reason for additional surgery in 17.5% of the patients, consistent with literature emphasising the importance of monitoring and workup for recurrent symptoms (Pecoraro et al. 2021). The need for ostomy creation at 14.3% was in keeping with the literature, highlighting complications requiring further surgical management (Munnangi et al. 2023; Quiroz et al. 2020). These findings highlight the complexity of managing HD and ongoing challenges post-initial surgery, stressing long-term follow-up and vigilance to optimise patient outcomes. It also emphasises the importance of continued research into strategies to minimise redo surgeries and improve overall surgical outcomes in HD patients.

There was a notable incidence of short-term complications (61.9%) following surgery for HD, with rates exceeding some of those previously reported, including South African and low-resource setting studies (11% – 57%) (Cantone et al. 2023; Mabula et al. 2014; Munnangi et al. 2023; Quiroz et al. 2020; Short, Durham & Rollins 2022; Zbaida et al. 2021). Postoperative ileus (18.0%) was the most common complication, which was higher than findings reported in recent literature (3.4% – 11%) (Wang et al. 2023; Zhang et al. 2023). This adds up to the overall complication rate and could be because of this study’s over-diagnosis of short-term ileus.

The researchers observed a high long-term complication rate (65.1%), significantly higher than those reported elsewhere (16% – 35.5%), suggesting a substantial impact on patient outcomes (Saleem et al. 2023). Approximately 37% of the patients developed enterocolitis, aligning with other research elsewhere and a South African study (16% – 50%), emphasising its role as a major postoperative concern (Arafa et al. 2022; Moore, Albertyn & Cywes 1996; Quiroz et al. 2020; Saleem et al. 2023; Wang et al. 2023). Similarly, hypomotility issues were reported in 36.6% of the cases, highlighting the frequency of functional complications after surgery (16.1% – 46.0%) (Quiroz et al. 2020; Saleem et al. 2023). These results emphasise the necessity for continuous monitoring and effective management strategies to mitigate long-term challenges, such as enterocolitis and hypomotility, in the care of patients with HD (Saleem et al. 2023).

The follow-up rate of 27.7% reflected the known challenges of long-term patient tracking similar to those reported in previous research from low-recourse settings (Mabula et al. 2014). Among the patients who adhered to follow-up appointments, a high survival rate of 95.7% was observed, on the higher side when compared to studies from low-resource settings (78.2% – 100%) (Ada et al. 2021; Bandré et al. 2010; Beltman et al. 2022; Bradnock et al. 2017; Mabula et al. 2014; Tan et al. 2022). The mortality rate among followed-up patients was 4.3%, lower than in studies from low-resource settings (6.7% – 21.8%) (Ada et al. 2021; Bandré et al. 2010; Beltman et al. 2022; Bradnock et al. 2017; Mabula et al. 2014). The median duration of follow-up was approximately 2 years, consistent with other South African and low-recourse setting studies and underscoring the importance of prolonged monitoring of outcomes (from 8 months to 6 years) (Mabula et al. 2014; Zbaida et al. 2021). These results aligned with current literature and emphasised the ongoing challenges of patient follow-up and the continued success of surgical interventions in enhancing survival rates and reducing mortality because of HD.

### Healthcare challenges and proposed solutions

Managing HD in resource-limited settings presents several challenges that have an impact on outcomes. Suboptimal delayed diagnosis due to misdiagnosis, socioeconomic barriers and limited awareness may increase the need for multistage procedures and risk of complications, highlighting the need for improved healthcare provider training, community education and newborn screening. Limited access to frozen section pathology and specialised tests delays diagnosis and definitive treatment, requiring investment in pathology infrastructure, telepathology and portable diagnostics. Many patients undergo staged procedures because of late presentation and bowel dilation, which could be reduced through early diagnosis. High postoperative complication rates, including enterocolitis, ileus and motility disorders, necessitate improved recovery protocols, standardised bowel management, patient education and structured follow-up. Loss to follow-up remains a concern, emphasising the need for mobile health solutions, community health workers and decentralised follow-up systems. Addressing these challenges through targeted interventions can improve early detection, optimise surgical strategies and enhance long-term care.

### Study limitations

The retrospective study design and reliance on medical records might have resulted in missing or incomplete data collection. The geographical region and referral patterns of clinicians in the drainage area of the UAHC might not fully represent the diversity of clinical presentations and outcomes found in broader contexts. Furthermore, the absence of prospective monitoring and a control group impeded the ability to assess long-term outcomes and directly link results to specific interventions.

### Recommendations for future research

Further research is needed to improve HD diagnosis, management and long-term care in resource-limited settings. Prospective studies should assess functional and quality-of-life outcomes after different surgical approaches. Comparative research should identify the most effective techniques to reduce complications and enhance recovery. Genetic and epidemiological studies could aid early detection and targeted interventions. Investigating optimal follow-up strategies is essential for improving long-term patient monitoring and postoperative care. Multicentre collaborations can enhance data collection, standardise treatment, and improve global outcomes. Focused research in these areas will help reduce disparities and advance HD management.

## Conclusion

This study examined the clinical profile, management and outcomes of HD at a tertiary hospital in central South Africa. Resource limitations pose significant challenges. Delayed diagnosis, scarce diagnostic tools and socioeconomic barriers increased surgical complexity and complications. Findings mirrored other South African and low-resource studies. A high male predominance, late diagnoses multistage surgeries were common, the latter because of delayed presentation. Despite these challenges, survival rates were high among patients with follow-up. However, many patients were lost to follow-up. Postoperative complications, especially enterocolitis and bowel dysmotility, were frequent. Better perioperative care and structured follow-up are essential. Early diagnosis, improved diagnostics and optimised surgical strategies are critical. Training healthcare providers, expanding diagnostic access and educating patients could reduce the morbidity of HD. Future research should refine surgical techniques, develop cost-effective diagnostics and improve follow-up in resource-limited settings.
